# The Health Care Consequences Of Australian Immigration Policies

**DOI:** 10.1371/journal.pmed.1001960

**Published:** 2016-02-16

**Authors:** John-Paul Sanggaran, Bridget Haire, Deborah Zion

**Affiliations:** 1 School of Public Health and Community Medicine, UNSW Australia, Sydney, Australia; 2 Kirby Institute, UNSW Australia, Sydney, Australia; 3 Victoria University, Footscray, Victoria, Australia

## Abstract

Deborah Zion and colleagues discuss the “dual loyalty” conflict affecting Australian health care providers involved in the care of asylum seekers.

**Summary Points**

- In Australia, immigration policy is to incarcerate those seeking asylum in order to deter others from coming.
- Within this environment, health care providers frequently experience “dual loyalty” conflict, whereby they cannot serve the interests of both their patients and their employers.
- The ratification of the Optional Protocol to the Convention Against Torture (OPCAT) would allow for domestic and international monitoring of places of detention, which would serve to ameliorate some of the most problematic aspects of the detention system, including the undemocratic lack of transparency.
- This would assist in resolving the “dual loyalty” conflict that health care workers must contend with in the current situation.

The displacement of people is an inevitable consequence of conflict, tyranny, and oppressive regimes worldwide. Those seeking asylum are acknowledged as among the most vulnerable of populations. Governments, however, are notoriously reluctant to accept large influxes of refugees.

Despite a global context in which people are being displaced in the highest numbers since the end of World War II [1], successive Australian governments have adopted policies explicitly intended to deter people from seeking asylum, including mandatory detention for indefinite periods of time in remote locations [2]. This policy consequentially erodes human rights protections afforded to people seeking asylum [3] and, indeed, to all people held under any form of imprisonment or detention [4], and we contend this creates specific problems for the ethical practice of medicine.

For physicians and other health care providers, the immigration detention context can result in a conflict of interest between (1) the government’s political imperative to apply rules that deter people from seeking asylum via boat and (2) the provision of acceptable medical care [5]. For example, doctors at mainland hospitals have been expected to treat asylum seeker children who would upon discharge be returned to detention centres. In October 2015, staff at the Royal Children’s Hospital in Victoria refused to do so, on the basis that it is unethical to discharge patients into a context that is unsafe and damaging to health [6].

Such situations, in which physicians and other health care providers are required to subordinate the interests of patients to those of the state or their employers, are characterised by Physicians for Human Rights as “dual loyalty conflicts” [7].

Under the current Australian policy, asylum seekers’ boats are turned back to their port of origin when possible, and when this is not possible, people on boats intercepted at sea are sent to offshore detention centres for indefinite periods [2].

People who have arrived by boat at these detention centres are refused any possibility of being settled in Australia, even if they are accorded refugee status [2]. Within these detention centres, health care delivery is outsourced to the private sector via a local subsidiary of an international company. This company is contractually obligated to deliver health care at a standard appropriate for Australia [8]. Documented failures to achieve such a standard are numerous, however, and include instances of asylum seekers being required to undergo health assessments while exhausted, dehydrated, and filthy, with clothing soiled by urine and faeces; artificial and dangerous delays in transfer of patients for tertiary care [9–11]; confiscation and destruction of medications, medical records, and medical devices [9–11], and individuals being addressed by boat number rather than name, according to multiple concordant eyewitness accounts [9–12].

An independent audit of medical service provision conducted in offshore detention centres on Christmas Island in 2012 showed that only 29% of a population of 1,311 of patients saw a doctor within a reasonable time, that appropriate childhood vaccinations occurred in 7% of cases, and that adequate clinical records were kept in only 25% of cases [13]. Despite an expectation that these measures would be met in 95% of cases, the health care delivery company responded to the review by stating that the standards should be lowered, rather than its performance improved [13].

These failures to meet objective standards of decent patient care make it particularly important that people who are contracted to provide such care are able to speak freely about the shortcomings of the system and advocate for improvements. However, two new pieces of legislation have further eroded personal security for people confined in detention centres by increasing the license of private contractors to use “excessive force” against detainees and by enacting legal sanctions against workers in detention centres who speak up about the conditions they encounter.

The first piece of legislation is a proposed amendment to the Migration Act that provides a legal defence to security contractors, such as security guards, working within immigration detention if they use excessive force on the grounds that they feel it is justified [14].

Critics of the law argued that it gives detention centre contractors a greater level of legal immunity than police have in Australia, despite these contractors having significantly less training and much lower standards of accountability. A retired Supreme Court Judge stated that the amendment would make guards freer with their use of force, even to the point where “the guards [would be] authorised to beat asylum seekers in detention centres to death” [15].

A further legislative change already introduced is the Border Force Act 2015 [16]. This Act makes it an offence to record or disclose information obtained by a person in their capacity as an “entrusted person.” What constitutes an entrusted person is broadly defined to include doctors, nurses, psychologists, teachers, and humanitarian workers working within the immigration detention context or, indeed, any person that has contact with an asylum seeker even outside of an immigration detention centre, for conducting normal activities including advocating for patients and bringing to light inadequacies in the provision of medical care [17]. The penalty for breaking this silence is a two-year jail term [16].

Much of what is known about the treatment of asylum seekers in Australia comes from whistle-blowers [9–11] who would be criminalised under this legislation [13]. Of particular concern is the fact that doctors are required by their professional codes of ethics to “advocate that the health care environment remains patient-centred at all times” [18] and to “…ensure that you do not countenance, condone or participate in the practice of torture or other forms of cruel, inhuman, or degrading procedures…” [19].

Additionally concerning is the listing of the Border Force Act under the Telecommunication Act 1979, which grants border control authorities the power to "scrape" the communications of its employees, as well as journalists, asylum seekers, and others—accessing private electronic communications without the consent of the correspondents [20].

There are already instances of journalists being investigated by the Australian Federal Police after reporting on immigration detention [21], and a member of the Australian Senate was confirmed to have been spied on by a private company contracted to provide security by the government when she visited an offshore detention centre [22].

These measures to infiltrate communications and penalise whistle-blowers are particularly worrisome given mounting and unequivocal evidence of inhumane living conditions and substandard medical care within immigration detention centres [2,6,9–11,13,23,24].

In the view of many [17,25–29] the above measures amount to intimidation, particularly of medical practitioners who have specific ethical obligations to protect patients’ best interests [18,19].

The result of these measures has been described as an attempt to generate a “chilling effect”; that is, the fear of imprisonment will prevent health care workers from speaking out about substandard care [29,30].

Members of the medical community have rejected the terms of the new legislation, and responses have included an open letter by health professionals, humanitarian workers, and teachers challenging the government to prosecute them while they act to maintain their professional integrity and to advocate for adequate conditions in detention [29]. As such, these health care providers have chosen the most difficult response to a dual loyalty conflict by putting themselves at risk to make public the unacceptable restrictions upon medical care imposed by the new legislation.

There is further evidence of substandard treatment. The Australian Human Rights Commission recently concluded a National Inquiry into Children in Detention [11]. The Inquiry’s findings concord with other sources that also detail substandard and harmful care, child abuse, sexual abuse, and abuse of the disabled [2,6,9,10,13,23,24]. In the adult detainee population there is documented death through apparent medical neglect [23] and death through violence [31].

Accordingly, Australia has been found to be in breach of its obligations to the Refuge Convention and the Convention Against Torture, inhumane, and degrading treatment (UNCAT) by the United Nations Special Rapporteur on torture [32]. In addition, we contend, as does the Australian Human Rights Commission’s report [11], that the National Inquiry into Children in Detention provides ample evidence that Australia is in breach of the Convention on the Rights of the Child (UNCRC).

The Australian Government responded to the inquiry by attacking the integrity of the President of the Human Rights Commission (a statutory office holder) [33], using ongoing demonising rhetoric referring to asylum seekers as “illegals” [34], publishing more expensive propaganda in refugee source countries [35], continuing to spend an estimated $440,000 AUD annually per person detained offshore [36], strengthening the veil of secrecy over immigration detention using the legislative measures described above [14,16], and voting against making the reporting of child abuse mandatory [37].

The medical colleges and other organisations representing health care professionals have, over several years, put out many position statements criticising immigration detention. The latest Joint Statement from 15 medical and allied health bodies calls for the release of all children from detention [38]. However there has been no substantive action taken and the situation only appears to be worsening.

Australia’s “stop the boats” policy is being promoted as a quasi-humane solution to distressing media reports of “boat people” drowning en route to Australia, where they planned to seek asylum. The emphasis on deterrence—in particular, the assurance that asylum seekers who arrive by boat will not, under any circumstances, be settled in Australia—is meant to attack the business model of so-called “people smugglers” by removing any possibility of Australian residence. However, “people smugglers” do not create the conditions in which people willingly take to leaky boats to seek safety and refuge—persecution, conflict, and desperation are the root causes. Furthermore, there is now evidence that the Australian government has been paying “people smugglers” on route to return asylum seekers to Indonesia [39], thus seriously undermining the “business model” argument they themselves have put forward.

The political actions that have been set in place to support border protection policy have obvious serious consequences for the medical care that is provided. In this highly charged, rhetoric-filled atmosphere, it is vulnerable individuals fleeing persecution that pay the all-too human cost.

Considering the current political landscape, there appears to be little which might constitute amelioration, let alone a solution to the present situation. An exception to this bleak outlook is the ostensible bipartisan support for the Optional Protocol to the Convention Against Torture (OPCAT) [2], which Australia signed up to in 2009, but has not yet ratified. The OPCAT would require and facilitate domestic and international monitoring of places of detention, thereby increasing transparency.

In line with this aim, Australia’s peak health bodies have endorsed a joint statement calling for the ratification of the OPCAT as a matter of priority [2,40].

Refugees are a global concern and responsibility, and the problem of displaced people will not disappear. The fact that protecting human rights is sometimes difficult is no reason to abandon them. Human rights belong to all persons. At this time in history, those who have the privilege to be citizens of prosperous and peaceful countries have obligations to protect those less fortunate and to alleviate their suffering.

Patients’ health and well-being must be upheld as the primary duty of medical professionals, and in circumstances in which there are serious structural impediments to pursuing that goal, medical professionals must become advocates for change. A commitment to the right to health and the understanding that all rights are indivisible demands no less. At the most basic level, the problematic level of medical care occurring in Australian-funded offshore detention centres requires increased transparency, independent monitoring, and systems by which positive change can be implemented and evaluated. The current situation, whereby vulnerable people suffer and medical professionals are implicitly complicit in delivering substandard service, cannot be allowed to continue and most certainly should not be emulated in other countries.

## Abbreviations

OPCAT: Optional Protocol to the Convention Against Torture
UNCAT: United Nations Convention Against Torture, inhumane, and degrading treatment
UNCRC: United Nations Convention on the Rights of the Child
